# Measuring the Inequalities in the Distribution of Public Healthcare Resources by the HRDI (Health Resources Density Index): Data Analysis from 2010 to 2019

**DOI:** 10.3390/healthcare10081401

**Published:** 2022-07-27

**Authors:** Jieyu Zhao, Yuchen Yang, Katsuhiko Ogasawara

**Affiliations:** 1 Graduate School of Health Sciences, Hokkaido University, Sapporo 060-0812, Japan; jacqueline8367@gmail.com (J.Z.); abrongonge@gmail.com (Y.Y.); 2 Faculty of Health Sciences, Hokkaido University, Sapporo 060-0812, Japan

**Keywords:** healthcare service, equality, healthcare allocation, health resource

## Abstract

Background: In China, a developing country, the imbalance of development exists in different fields, and the inequalities in the distribution of healthcare services have garnered increasing attention. This study aimed to assess the healthcare services allocation and compare the latest distribution ratios of the essential healthcare indicators with the national requirement values announced by the government to research the level of healthcare development in China. Methods: Data were extracted from the Chinese Statistical Yearbook (2010–2019). The Healthcare Resource Density Index (HRDI) was used to evaluate equity in the demographic and geographical dimensions. The requirement values related to the ratio of doctors, nurses, and institution beds per thousand people were drawn from government documents. The data of healthcare serviceability indicators were compared with those requirements to check the situation of each province’s medical development. Results: From 2010 to 2018, there was a sustainable upward trend in government investment, however, a noticeable drop in the investment in northeast areas was seen. Although the HRDI of the institutions, beds, doctors, and nurses experienced some small fluctuations over the years, the developing areas in the middle-west areas had almost approached the level of developed east areas. There were only four provinces that met the requirements of the government in all three indicators (the ratio of institution beds, doctors, and nurses per thousand people). Conclusion: The equality of the distribution of healthcare services in China was unfair between the eastern and middle-western areas. The government launched the developing requirements and paid additional attention to narrowing the imbalance among different economic level regions to meet the needs of the local people. Although many provinces did not meet the requirements for medical resources in 2019, the distribution of healthcare services was approached relatively equitably countrywide.

## 1. Introduction

The equality of healthcare resource distribution is an important issue worldwide [1,2,3] The equality of the healthcare service is considered that there are not any differences in accessing health services among regions, even though there exist gaps in social, economic, geographical, power and prestige levels [4,5,6,7]. In particular, abundant studies have been conducted in developing countries [1,2,4,8,9,10]. In Brazil, a study was conducted to monitor inequalities in the health workforce for over 15 years and showed that the equalities had increased over time. Furthermore, the distribution of doctors and nurses was more unequally distributed across the country [2]. Proverbially, China is still a developing country, although there has been an incredible improvement over the decade. Many social services, especially health services, have been enhancing. The government is trying to improve the levels to meet the requirements of the people, including the healthcare service levels [11]. Although China has prominent people and land, economic development has gaps in different areas. Based on published studies, China had an imbalance distribution of health services among other areas and had gaps between urban and suburban areas [12]. A Chinese study showed that inequalities existed between urban and suburban areas due to an imbalance of economic levels and differences in land size and population [13,14]. Many studies have focused on equality research and used the Gini or Theil Indexes, popular methods in this field [15,16]. Nevertheless, the health resource density index (HRDI) method is also commonly used to assess the equality of health service resources in China [8,17]. This method was successfully used in national-level research, which showed that there were huge gaps in the health workforce distribution between the eastern and middle-west areas [8,17,18]. In addition, it has been used for provincial health resource assessment in many studies from China [8,17,19]. A study was conducted to evaluate the equality of healthcare resource allocation and compared the distribution of health institutions in 13 cities in the Jiangsu province, China, by this method [17]. In 2019, the Notice of the General Office of the State Council on Issuing the Outline for the Planning of the National Medical and Health Service System (2015–2020) was promulgated to improve the service availability, capacity, and efficiency. The governments of all provinces, autonomous regions and municipalities must implement it conscientiously [20]. That’s why it is necessary to study the current situation of healthcare resource distribution at the national level to examine the effect of this policy. This would be effective evidence to enable policymakers to make decisions or modify existing policies to balance developed and developing areas. The Theil and Gini indices consider the number of the population as an important factor [1,20], which are popular methods to assess the equality situation. It bases on the ratio of the resources to the population, the value could demonstrate the healthcare situation to support the researchers in identifying equality [21]. However, it cannot consider the area of the region’s land as a factor influencing the distribution and equality of healthcare resources. So, this study selects the HRDI method to evaluate that. It uses the density of healthcare resources to present the situation of their distribution [21]. This method considers the factors of the population and the land area. It is not an inevitable consideration that land is an important factor in the evaluation of equality in many large areas.

Published literature used this method focused on the provincial level to conduct studies but not on the national situation. So, this study aimed to evaluate the national healthcare resource distribution, which showed the health service equality level. This study extracted data from the five key health service factors over the decade from the national statistics departments. We assessed the distribution of healthcare resources via the HRDI formula and attempted to show the level of equality in health services in China. The Notice of the General Office of the State Council on Issuing the Outline for the Planning of the National Medical and Health Service System (2015–2020) was announced in 2015 and offered the requirement of the ratio of vital health resources to the population [22]. We compared the data from 2019 to the aim value from the government to determine whether each province met the requirements after development over five years (2015 to 2020).

## 2. Materials and Methods

### 2.1. Data Sources and Setting

Data were extracted from the Chinese Statistic Yearbook (2010–2019), which covered 31 provinces, autonomous regions, and municipalities separated into four groups: northeastern, eastern, central, and western regions. The northeastern region included Heilongjiang, Jilin, and Liaoning (three provinces). The eastern region included Beijing, Tianjin, Hebei, Shanghai, Jiangsu, Zhejiang, Fujian, Shandong, Guangdong and Hainan (10 provinces and municipalities). The central region included Shanxi, Anhui, Jiangxi, Henan, Hubei, and Hunan (six provinces). The western region included Inner Mongolia, Chongqing, Guangxi, Sichuan, Guizhou, Yunnan, Tibet, Shaanxi, Gansu, Qinghai, Ningxia and Xinjiang (12 provinces, autonomous regions, and municipalities). The data included the amount of investment, the number of health institutions and beds, and the ratio of doctors and nurses per 1000 people, which were used to calculate the value of the HRDI to evaluate the distribution and equality of healthcare resources. Of the five, three factors were compared with the national medical and health service system requirements, which were the ratio of doctors, nurses, and beds per 1000 people.

### 2.2. Statistical Analysis

The HRDI was proposed by Zheng Xiaohua to assess the healthcare resources’ distribution in 1993 [23]. Several studies were conducted to validate the method’s effectiveness [18,23]. The HRDI represented the influence of the population and geographical factors on the density and equality of healthcare resources, which could avoid the limitation of considering only a factor of the population [24,25]. The HRDI formula was [23,24]:

(1) $$HRDI=\sqrt{\frac{HR_{i}}{P_{i}}*\frac{HR_{i}}{A_{i}}}$$

*HR_i_*: health resource quantity of the ith region

*A_i_*: geography of the ith region

*P_i_*: population of the ith region

*HRDI*: the value of the health resources density index

HRDI is a commonly used method in China to study the distribution of resources [7,16,18,23]. Since the 90 s, many studies have focused on regions in China to assess the situation of healthcare resources and demonstrate their equality [26,27]. This study introduced land area as an important factor for the evaluation of equality, especially in counties like China with a relatively large land that influenced the fairness of health services. In addition, there were requirements for the ratio of doctors, nurses, and beds to per 1000 people, as announced by the government’s document. This study compared the data of each province by 2020 to the aim value, which showed the effect of medical service development from 2015 to 2019.

## 3. Results

### 3.1. The Results of the HRDI

**Figure 1:**
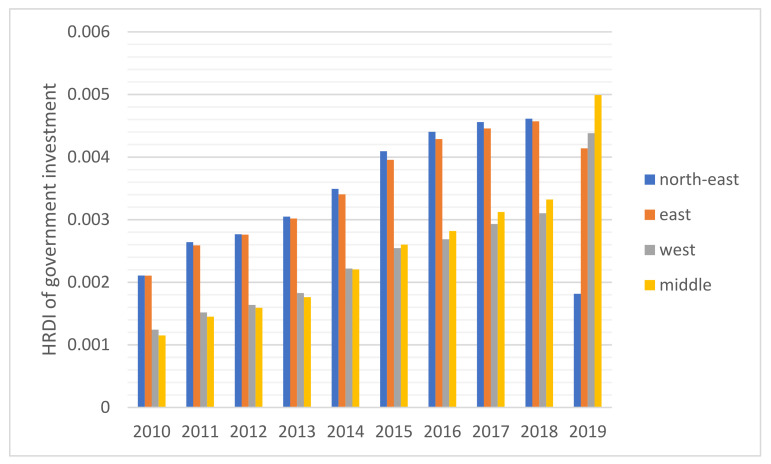
The HRDI of the health resources in investment when dividing China into four areas.

**Figure 2:**
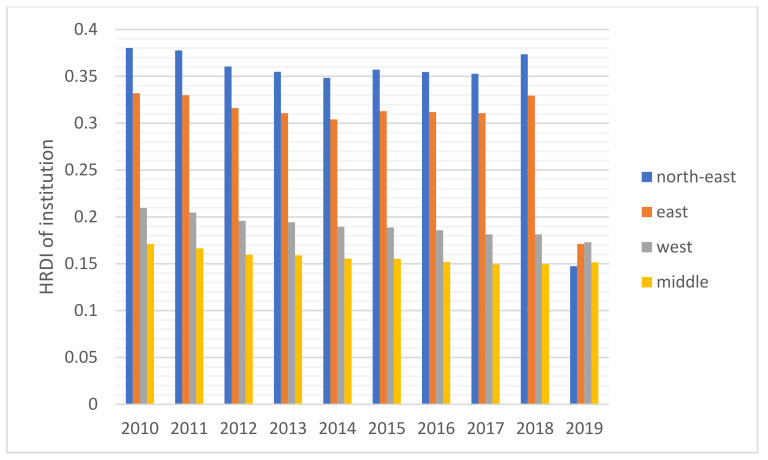
The HRDI of the health resources in institutions when dividing China into four areas.

**Figure 3:**
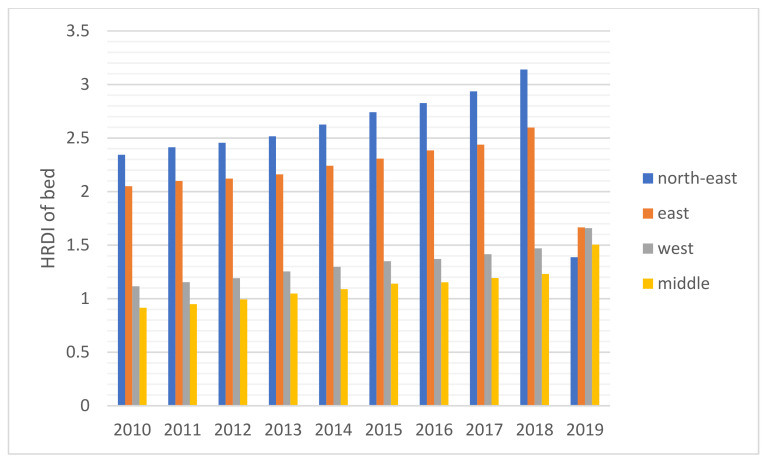
The HRDI of the health resources in beds when dividing China into four areas.

**Figure 4:**
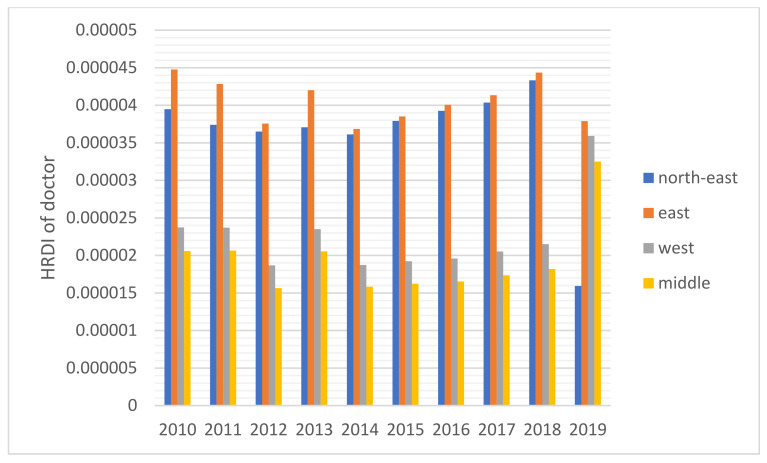
The HRDI of the health resources in doctors when dividing China into four areas.

**Figure 5:**
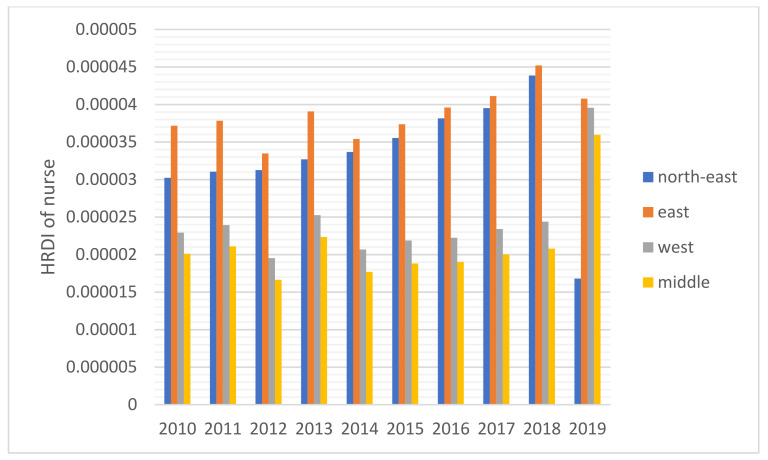
The HRDI of the health resources in nurses when dividing China into four areas.

The HRDI values of the healthcare resources in different areas are shown Figure 1, Figure 2, Figure 3, Figure 4 and Figure 5. For investment in Figure 1, the trends of the four areas were upward over the ten years. Rapid, sustained growth occurred in the northeast and east areas (approximately 0.002–0.0045), and modest growth occurred in the central and western areas (approximately 0.001–0.003). However, in 2019, the northeast region experienced an incredible downward trend from 0.0046 to 0.0018, more than a half of the value of 2018. Before 2019, the value of the middle and west density of the investment was less compared to that of the eastern areas, however, in 2019, the situation completely reversed. There were small fluctuations in the HRDI of the institutions between 2010 and 2018 in the northeast and east areas, as shown in Figure 2, and the value decreased to the lowest in 2014. The HRDI of the two areas was almost twice as large as the values in the western and middle areas. The trend of the HRDI in the central and western areas slightly decreased over ten years. It was not disregarded that the value of the institutions in the northeastern region and the eastern region had a marked drop from 0.37 in 2018 to 0.15 in 2019 and from 0.34 in 2018 to 0.17 in 2019, respectively. Meanwhile, in Figure 3, the situation of the HRDI regarding beds was similar to that of the institutions, except for a gradual increase from 2010 to 2018 in four areas. According to the doctors and nurses in Figure 4 and Figure 5, respectively, their trends were similar, while the density of the doctors was almost slightly greater than that of the nurses. In the first nine years, it was seen that the HRDI of the northeast areas and east areas was much greater than the western and central areas, especially in 2018 when the differences were maximum. In 2019, there were changes in the four areas. Eastern areas experienced a decrease in doctors and nurses from $4.4\times 10^{-5}$ to $3.8\times 10^{-5}$ and from $4.5\times 10^{-5}$ to $4.1\times 10^{-5}$, respectively. It was worth noting that numbers had fallen precipitously in the northeast area for both factors in 2019. However, there were dramatic increases in doctors and nurses in the two areas of western and middle areas. These values approached the HRDI value in the eastern area in 2019 (approximately $3.5\times 10^{-5}$ of doctors, $4.1\times 10^{-5}$ of nurses, respectively).

### 3.2. A Comparison with Requirements of the Government

**Figure 6:**
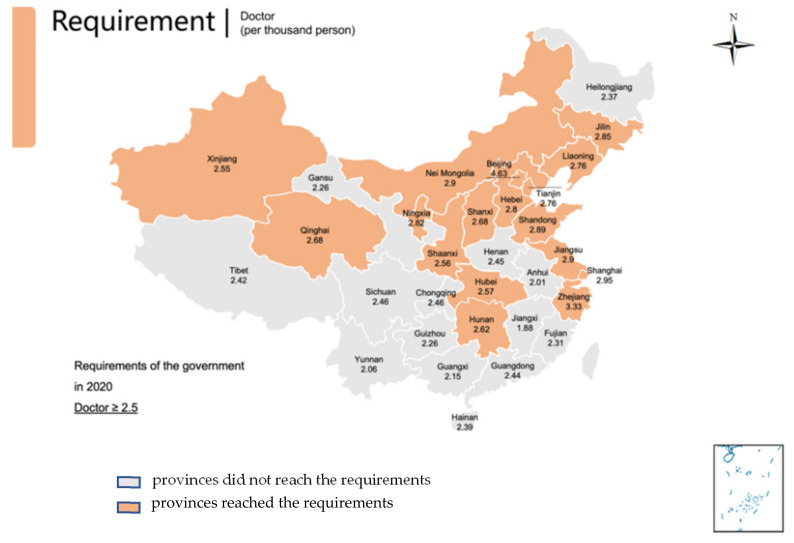
Provinces reached the requirements of the number of doctors in 2020 by the government; Provinces did not reach that requirement.

**Figure 7:**
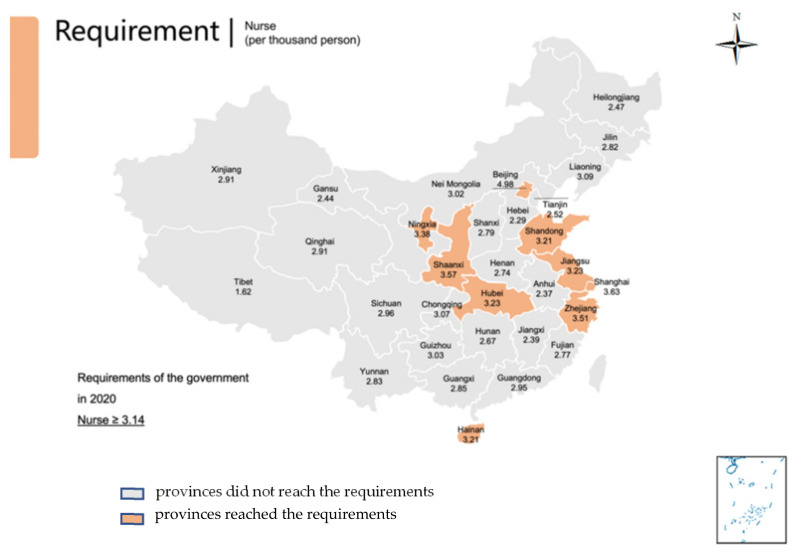
Provinces reached the requirements of the number of nurses in 2020 by the government; Provinces did not reach that requirement.

**Figure 8:**
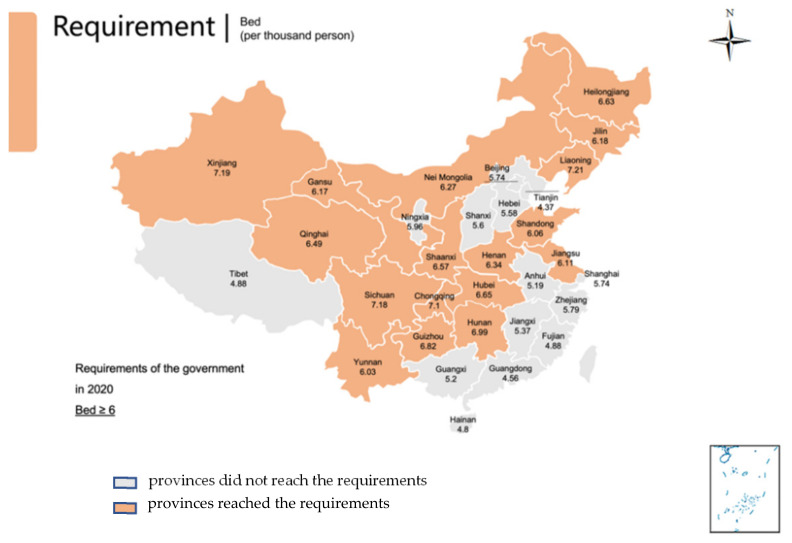
Provinces reached the requirements of the number of beds in 2020 by the government; Provinces did not reach that requirement.

The situation of the 31 provinces’ health services developments and each province’s ratio of doctors, nurses, and beds to 1000 people in 2019 is presented in Table 1 and Figure 6, Figure 7 and Figure 8 with the aim ratio of those from the government document. The values in the orange table cells and orange provinces reached the requirements, while the values in the white table cells and grey provinces did not. A total of 17 provinces (Liaoning, Jilin, Beijing, Tianjin, Hebei, Shanghai, Jiangsu, Zhejiang, Shandong, Inner Mongolia, Shaanxi, Qinghai, Ningxia, Xinjiang, Shanxi, Hubei and Hunan) met the requirement of at least 2.5 doctors per 100 people (Table 1, Figure 6). In addition, nine provinces met the requirement of at least 3.14 nurses per 1000 people (Beijing, Shanghai, Jiangsu, Zhejiang, Hainan, Shaanxi, Ningxia and Hubei) (Table 1, Figure 7). Furthermore, 17 provinces (Liaoning, Jilin, Heilongjiang, Jiangsu, Shandong, Inner Mongolia, Chongqing, Sichuan, Guizhou, Yunnan, Shaanxi, Gansu, Qinghai, Xinjiang, Henan, Hubei and Hunan) met the requirement of six beds per 1000 people (Table 1, Figure 8). Remarkably, only four provinces met all three indicator requirements (Jiangsu, Shandong, Shaanxi, and Hubei).

## 4. Discussion

From Figure 1, it is shown that the sum of the investment gradually increased, which means that the government took efforts into healthcare services and tried to narrow the gaps between the developed and developing areas. The eastern area was a developed region in China, hence the local government invested additional money and attention in the development of health services. However, the national government found economic development differences between the eastern and middle-western areas. Based on the national medical reform of 2009, investment increased in developing areas. A study also mentioned that a great deal of financial investment in the backward region promoted the level of local medical services sustainably to meet the people’s needs [19]. However, financial investment should match the developing economic speed. With sufficient medical institutions and adequate workforce and equipment, too much financial investment was unnecessary and could produce fiscal profligacy problems. As a result, from 2015, the investment in medical services gradually approached stability among the four areas.

For the HRDI of the institutions, beds, doctors, and nurses, from 2010 to 2018, although some more minor fluctuations were seen, the situation did not show a noticeable increase. In addition, the northeast and east areas had significant advantages over the western and central regions. There were many reasons for this. First, healthcare development did not catch up with the increasing speed of the population and could not meet the needs of the people. Second, medical education takes approximately eight to ten years, a long time to influence the number of medical workforces. Finally, it must be mentioned that the developed regions were more attractive to doctors and nurses than the developing areas. A study stated that China’s developed areas offered a higher salary for healthcare workers and a more comfortable living environment, such as children’s education and public traffic systems [12]. However, it was worthy of notice that the ratio of doctors and nurses increased significantly in the west and central areas in 2019 since the government launched several policies to encourage talented workforce and professionals to work there, such as offering financial subsidies and housing.

Based on the results of this study, additional attention should be paid to 2019 as the year experienced a new change in comparison to the previous years. Above all, the northeast area faced a dramatic drop in the four indicators, and the fall reached almost 50%. Owing to decreased GDP in this area, local people were prone to find work positions in other better-developing regions that caused a significant loss of population [28]. Furthermore, the northeast area, as a heavy industry base, met the shifting industry structure since the Chinese government recently considered environmental protection. The ratio of institutions and beds with a gradual decline cannot be disregarded in the Eastern region, caused by the government’s enhancement of management of medical institutions. Furthermore, it was also due to closing a part of the institutions that did not meet the new regulations of the government and combining small-sized institutions to improve healthcare service efficiency.

The inequality of healthcare services has been gradually decreasing, which was found in this study. Another research with similar results introduced the situation from 2013 to 2017 [8]. The east area, as a developed region, has the best level of allocation of medical resources, which was also found by a study [8]. It is worthy to notice that the allocations of the east, west and central region are beginning to converge, which means the healthcare service has been becoming closely equal. That probably resulted from the “Comprehensive reform of public hospitals” [29]. It was announced by the National Health Commission (NHC) of the People’s Republic of China in 2017. That includes that NHC should make the number and scale of the public hospitals reasonable and enhance the responsibility for the management of that [29]. In China, public hospitals play a key role in Chinese healthcare service and make up the majority of hospitals. That means the public hospital’s distribution almost decides the equality level of medical service. That’s why there is an evident change in 2019.

Regarding the requirements of the government, only four provinces met the needs of all three indicators in 2019. However, large regions of the developing areas, such as the western and central regions, did not reach these aims. It is worth noting that there was a considerable gap between the nurse number and other factors. That means the resources of nurse’s severe shortage in China. Therefore, the government should train more personnel and encourage them to service patients by enhancing the workplace situation and salary. The doctor’s position was better than the number of nurses, but the problem needs to improve in the central, west, and northeast areas. The bed number met the government’s requirement in most places, especially in relatively poor areas, but some developed regions lacked beds. That means the government invested in these regions to make the facilities sufficient. In the developed region, the number of beds cannot meet the requirement of the government and the people, as these areas are dense people.

This study decided to use the HRDI method rather than other more popular methods, such as the Gini and Theil Indexes. Since the distance travelled between the healthcare institution and residents was a key factor, for example, the land of western area in China had large land; however, with a less population, the convenience of commuting to healthcare institutions should be taken into consideration. The number of institutions, doctors, and nurses in a region demonstrated this situation. As a country with a large landmass, the influence of the land should not be ignored as important as the population [17]. However, most published studies just researched the provincial data, not national data. Furthermore, most studies that used this method were published in Chinese, which caused fewer studies from other countries can be referred to.

Equality assessment is an effective method to study healthcare service capacity, especially in countries that are developing procedures to reduce inequality among different regions. In China, since 2009, the government found that an imbalance of developments and medical services could not meet the local people’s needs to launch a series of reform policies [17]. Based on this study’s results, healthcare service promotions were found, and the effects of government reforms were presented. However, policymakers should be reminded that several provinces did not meet the government’s requirements. In the future, the government should take a relatively backward step to consider and look out for those areas’ lack of medical resources. Additionally, as they progress, the middle-western regions should maintain healthcare serviceability by developing an economic situation to offer stable and comfortable living for the healthcare workforce.

This study focused on the national equality of healthcare service allocation and selected the HRDI method that corresponds with the Chinese population and geography situation. Less national research is considered a factor, but necessary. This study tried to offer more powerful evidence about that. Additionally, the central government made a requirement of medical resource distribution. This study verified each province’s result, offering direct evidence of development. The most recent published studies used HRDI to assess the distribution of healthcare resources, almost at the provincial level, but, not at the entire national level. Hence, there were limited related papers to compare and analyze our results against. The HRDI was different from the Theil Index and had an expected value to assess the equality level, however, it only compared the given areas to evaluate the distribution situation. This method is useful to conduct the overall picture of distribution in an area, especially countries with large land.

## 5. Conclusions

The equality of the distribution of healthcare services in China was unfair between the eastern and middle-western areas. The government launched the developing requirements and paid additional attention to narrowing the imbalance among different economic level regions to meet the needs of the local people. Although many provinces did not meet the requirements for medical resources in 2019, the distribution of healthcare services was approached relatively equitably countrywide. The lack of nurses should be paid more attention to by the government. In the future, a deep study on the Chinese distribution of healthcare resources should be conducted, especially, focusing more on the difference between urban and rural regions. The existing inequality among areas should be discovered. Probable reasons contributed to that should be investigated.

## Figures and Tables

**Table 1:** The requirements of a policy from the government, compared to each province’s indicators.

Area	Province	Doctor (per Thousand Person)	Nurse (per Thousand Person)	Bed (per Thousand Person)
North-east	Liaoning	2.76	3.09	7.21
North-east	Jilin	2.85	2.82	6.18
North-east	Heilongjiang	2.37	2.47	6.63
East	Beijing	4.63	4.98	5.74
East	Tianjin	2.76	2.52	4.37
East	Hebei	2.8	2.29	5.58
East	Shanghai	2.95	3.63	5.74
East	Jiangsu	2.9	3.23	6.11
East	Zhejiang	3.33	3.51	5.79
East	Fujian	2.31	2.77	4.88
East	Shandong	2.89	3.21	6.06
East	Guangdong	2.44	2.95	4.56
East	Hainan	2.39	3.21	4.8
West	Nei Mongolia	2.9	3.02	6.27
West	Guangxi	2.15	2.85	5.2
West	Chongqing	2.46	3.07	7.1
West	Sichuan	2.46	2.96	7.18
West	Guizhou	2.26	3.03	6.82
West	Yunnan	2.06	2.83	6.03
West	Tibet	2.42	1.62	4.88
West	Shaanxi	2.56	3.57	6.57
West	Gansu	2.26	2.44	6.17
West	Qinghai	2.68	2.91	6.49
West	Ningxia	2.82	3.38	5.96
West	Xinjiang	2.55	2.91	7.19
Center	Shanxi	2.68	2.79	5.6
Center	Anhui	2.01	2.37	5.19
Center	Jiangxi	1.88	2.39	5.37
Center	Henan	2.45	2.74	6.34
Center	Hubei	2.57	3.23	6.65
Center	Hunan	2.62	2.67	6.99
Requirements of the government in 2020		Doctor	Nurse	Bed
		≥2.5	≥3.14	≥6

## Data Availability

Data were extracted from the Chinese Statistic Yearbook (2010–2019). https://www.chinayearbooks.com/ (accessed on 1 June 2022).
